# Antidromic atrioventricular reentrant tachycardia in a patient with Wolff‐Parkinson‐White syndrome and unapparent preexcitation: A case report

**DOI:** 10.1002/joa3.12634

**Published:** 2021-09-10

**Authors:** Ayano Enzan, Sou Takenaka, Akihiko Ueno, Masayoshi Sakakibara, Terumasa Koyama, Shiro Uemura

**Affiliations:** ^1^ Division of Cardiology Kawasaki Medical School Kurashiki Japan; ^2^ Department of Cardiology IMS Katsushika Heart Center Tokyo Japan

**Keywords:** ablation, accessory pathway, antidromic atrioventricular tachycardia, WPW syndrome

## Abstract

This is a case of antidromic AVRT in a patient with unapparent preexcitation, and we could successfully diagnose and treat with the careful interpretation of wide QRS tachycardia. We should keep in mind that differentiation between intermittent and unapparent preexcitation is difficult, and some patients with unapparent preexcitation have short refractory periods of those accessory pathways, leading to sudden death.

## Graphical abstract

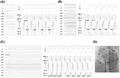

## 1 CASE PRESENTATION

**FIGURE 1:**
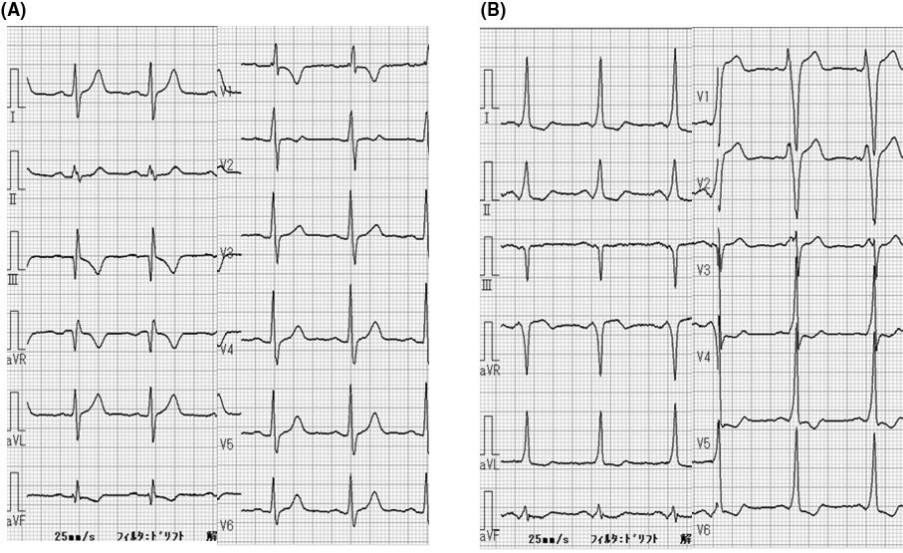
Twelve‐lead surface electrocardiogram before the procedure. The absence (A) and presence (B) of anterograde conduction through an accessory pathway

A 50‐year‐old man with WPW syndrome with unapparent ventricular preexcitation has been suffering from palpitating attacks since his teenage years (Figure 1). He experienced tachycardia that lasted <15 minutes and stopped with breath holding. Thus, his electrocardiogram (ECG) at the time of the attack was not captured.

A 12‐lead ECG at the time of entering the catheter room transiently recognized ventricular preexcitation. An electrophysiological study was conducted using decapolar catheters positioned into coronary sinus (CS) and the His bundle and a quadripolar catheter positioned at the right ventricle.

**FIGURE 2:**
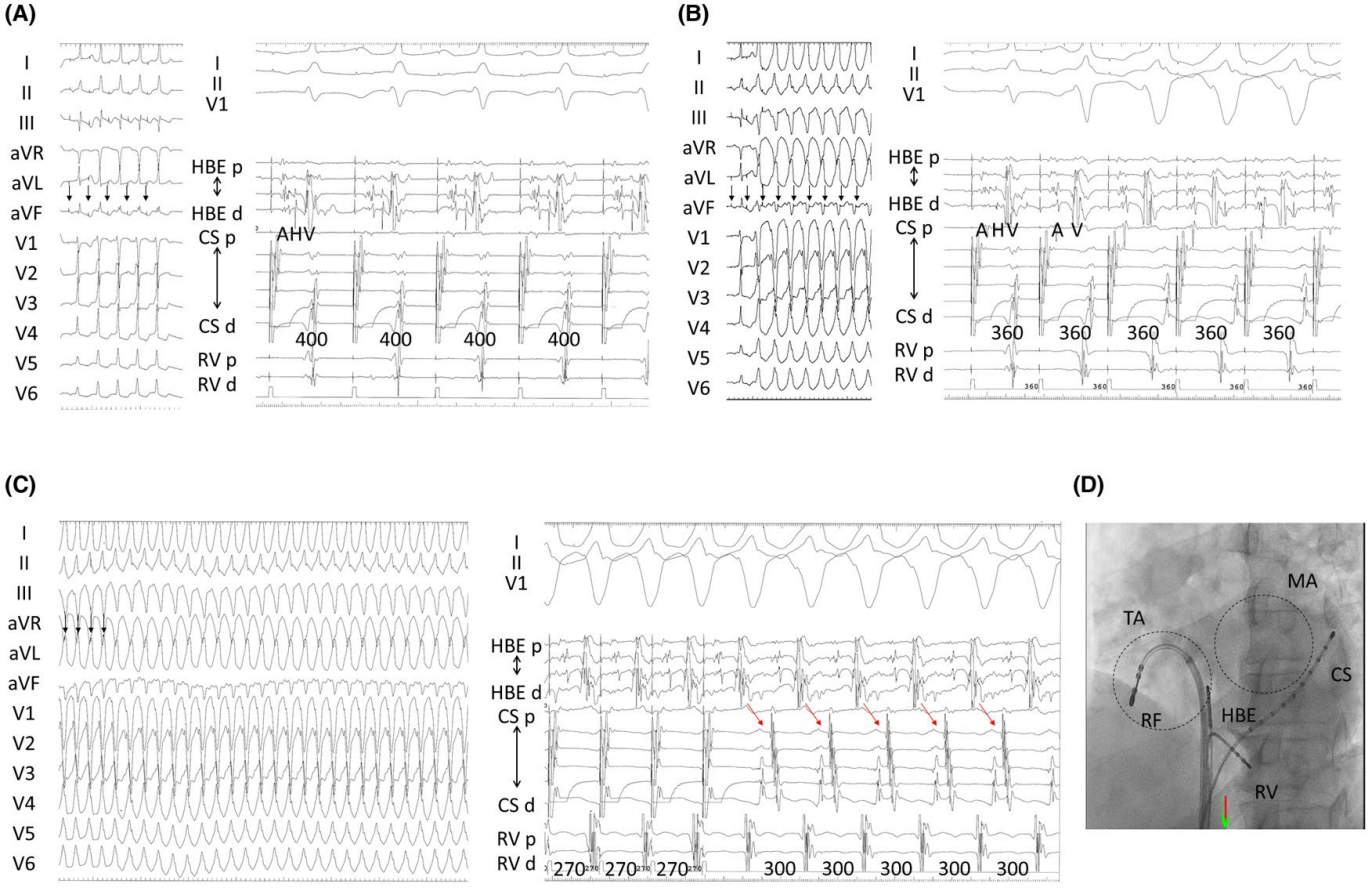
(A–(C) The left panels showed 12‐lead electrocardiograms during pacing (pacing spike = black arrow), and the right panels showed intracardiac recordings. A, Burst pacing of 400 ms from the distal portion of CS. QRS complex was narrow and atrioventricular (AV) conduction was via AV node. B, Burst pacing of 360 ms from the distal portion of CS. QRS complex became wide, and the intracardiac recordings of the activation of the bundle of His revealed no visible His potential. C, Burst pacing of 270 ms from the distal portion of CS. Wide QRS tachycardia (TCL = 300 ms) was induced by this pacing. The earliest site of ventricular atrial conduction was near the CS ostium (red arrow). QRS complex caused by a pacing of 270 ms was similar to this tachycardia. D, The catheter positioning in this study. Initially, a decapolar catheter was placed into CS. Dot circles show MA and TA. Abbreviations: CS, coronary sinus; HBE, bundle of His; MA, mitral annulus; RF, ablation catheter; RV, right ventricle; TA, tricuspid annulus

Ventricular preexcitation disappeared with burst pacing of 500 ms from CS and was not observed even with 400 ms (Figure 2A). Wide QRS tachycardia occurred with reproducibility with a pacing of 360 ms (Figure 2B) applied.

**FIGURE 3:**
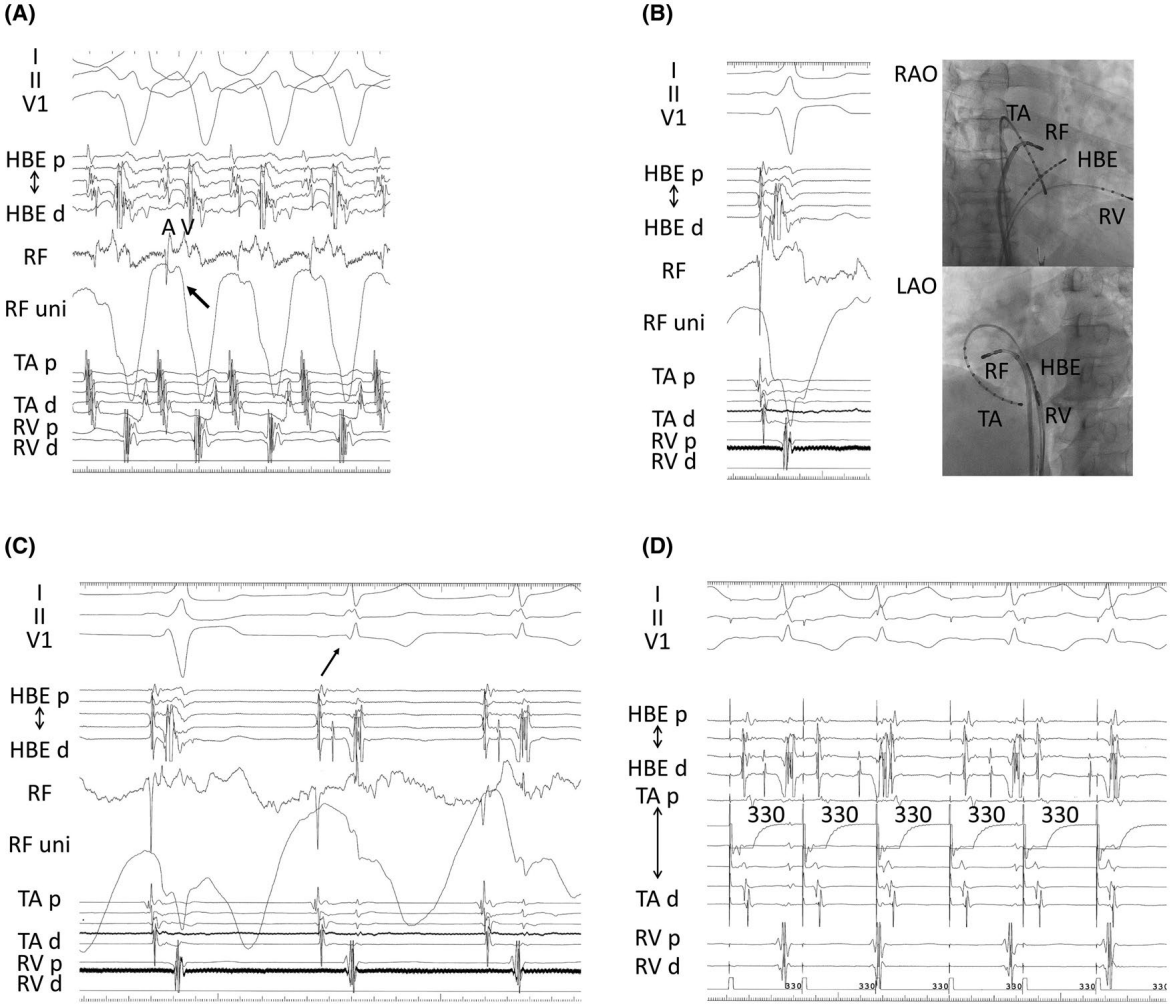
A, The intracardiac recording during this tachycardia. The ablation catheter was located at the lateral site of TA. The unipolar of the RF catheter showed the QS pattern (black arrow). B, The intracardiac recording during sinus rhythm (left panel) and the catheter position at the ablation (right panel). Additionally, the unipolar showed the QS pattern. The decapolar catheter was placed along with TA. C, The intracardiac recording during the application energy. QRS complex changed from wide to narrow (black arrow), and the conduction over the AV accessory pathway was eliminated. D, The intracardiac recording of a pacing of 330 ms. Any tachycardia could not be induced by a pacing maneuver. Abbreviations: HBE, bundle of His; RF, ablation catheter; RV, right ventricle; TA, tricuspid annulus

The tachycardia was induced using atrial burst pacing. QRS complex in tachycardia was the same in maximally preexcited QRS during atrial burst pacing of ≤360 ms (Figure 2C). The 1:1 antegrade accessory pathway (AP) conduction was observed with burst pacing of 270 ms. The earliest ventricular site during this tachycardia was the tricuspid annulus free wall (Figure 3A,B).

The earliest site of the ventriculoatrial (VA) conduction was near the CS ostium (Figure 2C). Moreover, a one‐to‐one VA conduction (Figures 2C and 3A) was noted. Intracardiac recordings of the activation of the His bundle revealed invisible His potential during tachycardia (Figures 2C and 3A). The unipolar electrode of local potentials at the earliest site showed the QS pattern (Figure 3A,B). This tachycardia was consistent with antidromic atrioventricular reentrant tachycardia (AVRT) using the right‐side free wall AP. Any tachycardia could not be induced by the pacing maneuver after eliminating this AP (Figure 3C,D). Additionally, he was free from palpitation.

## 2 DISCUSSION

In his 12‐lead ECGs before the procedure, there were times when preexcitation was present and times when it was absent. From his 12‐lead ECG with preexcitation, the location of his AP was located in RA freewall. Initially, we guessed the refractory period of his AP would be long, and his preexcitation would be intermittent. However, pacing with short PCL (≤360 ms) not only repredicted preexcitation but also induced antidromic AVRT. The mechanism of unapparent preexcitation is dominant conduction down the AV node.

This tachycardia was too fast, so we did not perform ventricular pacing during tachycardia, and failed to perform septal A synchronous premature atrial complexes timed to His refractoriness. Therefore, we could not conclusively exclude atrial tachycardia or AV nodal reentrant tachycardia with a bystander AP. Node‐ventricular Mahaim fiber has decremental conduction properties without the strong AV conduction, and slow AV conduction time. The cycle length of this tachycardia was too fast, and the AV interval time at the success site was short. The existence of Mahaim fiber was negative. Additionally, any tachycardia could not be induced after eliminating AP conduction.

The one‐to‐one antegrade AP conduction was observed using burst pacing of 270 ms. The onset of AF may shift to ventricular fibrillation, causing sudden death, in patients with very fast conduction over AP. Some patients with unapparent WPW syndrome have this phenomenon, which can lead to sudden death.

## CONFLICT OF INTEREST

Authors declare no conflict of interests for this article.

